# Broadening horizons: the contribution of mitochondria-associated endoplasmic reticulum membrane (MAM) dysfunction in diabetic kidney disease

**DOI:** 10.7150/ijbs.86608

**Published:** 2023-08-21

**Authors:** Yong Liu, Yingjin Qiao, Shaokang Pan, Jingfang Chen, Zihui Mao, Kaidi Ren, Yang Yang, Qi Feng, Dongwei Liu, Zhangsuo Liu

**Affiliations:** 1 Research Institute of Nephrology, Zhengzhou University, the First Affiliated Hospital of Zhengzhou University, Zhengzhou 450052, P. R. China.; 2 Traditional Chinese Medicine Integrated Department of Nephrology, the First Affiliated Hospital of Zhengzhou University, Zhengzhou 450052, P. R. China.; 3 Henan Province Research Center for Kidney Disease, Zhengzhou 450052, P. R. China.; 4 Key Laboratory of Precision Diagnosis and Treatment for Chronic Kidney Disease in Henan Province, Zhengzhou 450052, P. R. China; 5 Blood Purification Center, the First Affiliated Hospital of Zhengzhou University, Zhengzhou 450052, P. R. China.; 6 Department of Pharmacy, the First Affiliated Hospital of Zhengzhou University, Zhengzhou 450052, P. R. China.; 7 Clinical Systems Biology Laboratories, the First Affiliated Hospital of Zhengzhou University, Zhengzhou, 450052, P. R. China.

**Keywords:** Mitochondria-associated endoplasmic reticulum membrane (MAM), diabetic kidney disease (DKD), mitochondrial physiology, calcium homeostasis, lipid metabolism

## Abstract

Diabetic kidney disease (DKD) is a global health issue that presents a complex pathogenesis and limited treatment options. To provide guidance for precise therapies, it is crucial to accurately identify the pathogenesis of DKD. Several studies have recognized that mitochondrial and endoplasmic reticulum (ER) dysfunction are key drivers of the pathogenesis of DKD. The mitochondria-associated ER membrane (MAM) is a dynamic membrane contact site (MSC) that connects the ER and mitochondria and is essential in maintaining the normal function of the two organelles. MAM is involved in various cellular processes, including lipid synthesis and transport, calcium homeostasis, mitochondrial fusion and fission, and ER stress. Meanwhile, recent studies confirm that MAM plays a significant role in the pathogenesis of DKD by regulating glucose metabolism, lipid metabolism, inflammation, ER stress, mitochondrial fission and fusion, and autophagy. Herein, this review aims to provide a comprehensive summary of the physiological function of MAMs and their impact on the progression of DKD. Subsequently, we discuss the trend of pharmaceutical studies that target MAM resident proteins for treating DKD. Furthermore, we also explore the future development prospects of MAM in DKD research, thereby providing a new perspective for basic studies and clinical treatment of DKD.

## Introduction

Diabetes mellitus (DM) is a prevalent global health problem with approximately 10% morbidity 1. Epidemiological statistics show that the number of diabetic patients is projected to reach 537 million by 2021 and is expected to increase to 783 million by 2045^2^, ^3^. Diabetic kidney disease (DKD), a chronic renal microvascular disease caused by DM, has been considered the major cause of end-stage renal disease (ESRD) 4. It is estimated that approximately 30-40% of patients with DM will develop DKD 5, and the prevention and treatment of DKD has become a global health problem 6. DKD is characterized by a decrease in the glomerular filtration rate (GFR), fusion of podocytic foot processes, mesentery proliferation, and thickening of the glomerular basement membrane. It is also accompanied by albuminuria, renal inflammation, and tubulointerstitial fibrosis. The pathogenesis of DKD is very complex, and only limited therapeutic options are available 7. Although the progression of DKD can be delayed by inhibiting the continuous rise of blood sugar, blood pressure and lipid levels, some DKD patients still experience severe and progressive renal injury due to either untimely treatment or insensitivity to medication, which may ultimately lead to ESRD 8-10. Therefore, there is a pressing need to expand our understanding of the pathogenesis and identify promising targets for the prevention and treatment of DKD. Renal hemodynamic changes, glucose and lipid metabolism disorders, inflammatory responses, and oxidative stress (including mitochondrial damage and endoplasmic reticulum stress) are generally considered the primary causes of DKD 11, 12.

During the past few years, the important role of organelles in human diseases has been widely studied. Maintaining organelle homeostasis is crucial for living cells, but organelles are not isolated structures. Organelles facilitate material exchange and signal transduction among each other through membrane junctions and vesicle transport, which can have synergistic or antagonistic effects. With the in-depth study of organelles, many studies have shown that mitochondrial dysfunction and endoplasmic reticulum (ER) stress play key roles in the development of DKD 13, 14. The mitochondrial-associated endoplasmic reticulum (MAM) is a dynamic membrane contact site (MCS) between the mitochondria and ER that helps maintain their normal function 15, and this crucial connection has been found to play a pivotal role in the development of DKD 16.

Intercellular communication plays a crucial role in maintaining cellular homeostasis and overall organism health. Both direct communication of MCSs and vesicle transport are essential for this process. MCS refers to areas where intracellular membrane compartments are in close proximity, but membrane fusion does not occur 17. In these regions, the protein frenulum facilitates tight fitting of the membranes of the two organelles, enabling fast, direct, and mutual signaling between them 18. MAM is the first discovered physical link between two intracellular organelles 19, 20. The intricate relationship between MAM and cellular function has been the subject of extensive research in recent years, with new insights emerging into their role in various physiological and pathological processes. Recent research has revealed that MAM plays crucial roles in various cellular processes, including Ca^2+^ signal transduction, inflammation, lipid metabolism, ER stress, autophagy, and apoptosis 15.

## Structure and molecular composition of MAM

**Figure 1:**
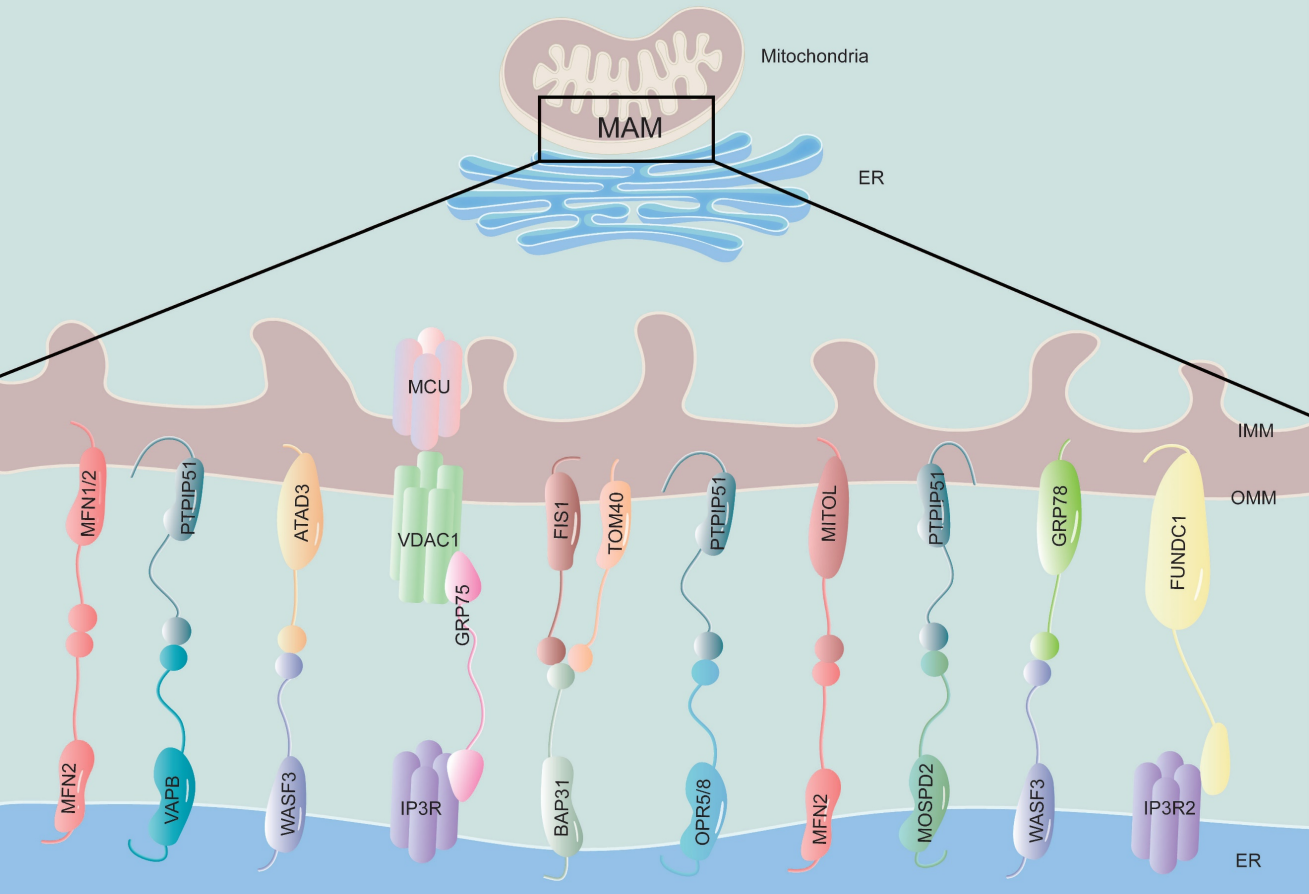
** The structure and components of MAM**. The physical connection region between the ER and mitochondria is called the MAM, which is composed of the mitochondrial outer membrane and the endoplasmic reticulum membrane. This structure is rich in various proteins and is connected by tethering proteins, which include MFN2-MFN1/2, VAPB-PTPIP51, WASF3-ATAD3, IP3R-GRP75-VDAC1, BAP31-FIS1, OPR5/8-PTPIP51, MFN2-MITOL, MOSPD2-PTPIP51, WASF3-GRP78 and FUNDC1-IP3R2. MFN1/2, mitofusin 1/2; VAPB, vesicle-associated membrane-protein-associated protein B; PTPIP51, protein tyrosine phosphatase-interacting protein 51; WASF3, Wiskott-Aldrich syndrome protein family member 3; ATAD3, ATPase family AAA Domain-containing protein 3; IP3R, inositol 1,4,5-trisphosphate receptor; GRP75, glucose regulate protein 75; VDAC1, voltage-dependent anion channel 1; BAP31, B-cell receptor-associated protein 31; FIS1, mitochondrial fission 1 protein; OPR5/8, oxysterol-binding protein related-protein 5 and 8; MITOL, mitochondrial ubiquitin ligase; MOSPD2, motile sperm domain-containing protein 2; GRP78, glucose regulate protein 78; FUNDC1, FUN14 domain-containing protein 1.

The ER is tightly attached to 5-20% of the mitochondrial surface, forming a specialized ER domain known as the MAM 21 (**Figure 1**). The close membrane association between the ER and mitochondria was initially observed using electron microscopy in the 1950s 22. With the advent of advanced imaging technology, the existence of MAMs has been confirmed using various techniques, such as real-time imaging, new electron microscopy methods, and subcellular isolation 23. MAM can be isolated through proteolytic hydrolysis, revealing a protein bridge that connects the outer mitochondrial membrane (OMM) and the ER. Electron microscopy studies have confirmed the existence of this bridge 24. This close association between the two organelles facilitates the exchange of lipids, calcium ions, and other signaling molecules. For an MAM to function properly, it is important to maintain a distance between the ER and OMM of no less than 10 nm and no more than 30 nm 25. This distance is necessary for efficient protein interaction and material exchange. If the distance between the mitochondria and ER is too close, less than 7 nm, or too wide, greater than 50 nm, it can lead to dysfunction in the MAM 26.

The MAM acts as a bridge between the ER and mitochondria and relies mainly on proteins to carry out its functions. Proteomics technology has identified hundreds to thousands of proteins in MAMs across various species and tissues, with highly conserved components 27-29. A recent proteome analysis of MAM in diabetic and nondiabetic mouse brains revealed significant changes in 144 proteins due to DM 30, indicating that components of MAM are altered during the process of disease. Studies have increasingly found newly discovered MAM-related proteins, and their functions are being gradually revealed. The flexibility of MAM protein assemblies allows for the recruitment of different signaling molecules based on specific cell states 31, 32. Resident proteins on the MAM are classified according to their different functions, for example, Ca^2+^ transport-related protein: inositol 1,4,5-trisphosphate receptor (IP3R) 33; lipid synthesis and transfer-associated protein: phosphatidylserine synthase 1 and 2 (PSS1/2) 34; mitochondrial dynamic regulatory proteins: mitofusin 2 (MFN2), dynamin-related protein 1 (DRP1) 35; insulin signaling-related protein: protein kinase B (PKB), mammalian target of rapamycin complex (mTORC) 36; and so on. The presence of multifunctional proteomes in MAM indicates that they exert crucial roles in regulating cellular homeostasis and biological processes (**Table 1**).

## Key physiological functions of MAMs

**Figure 2:**
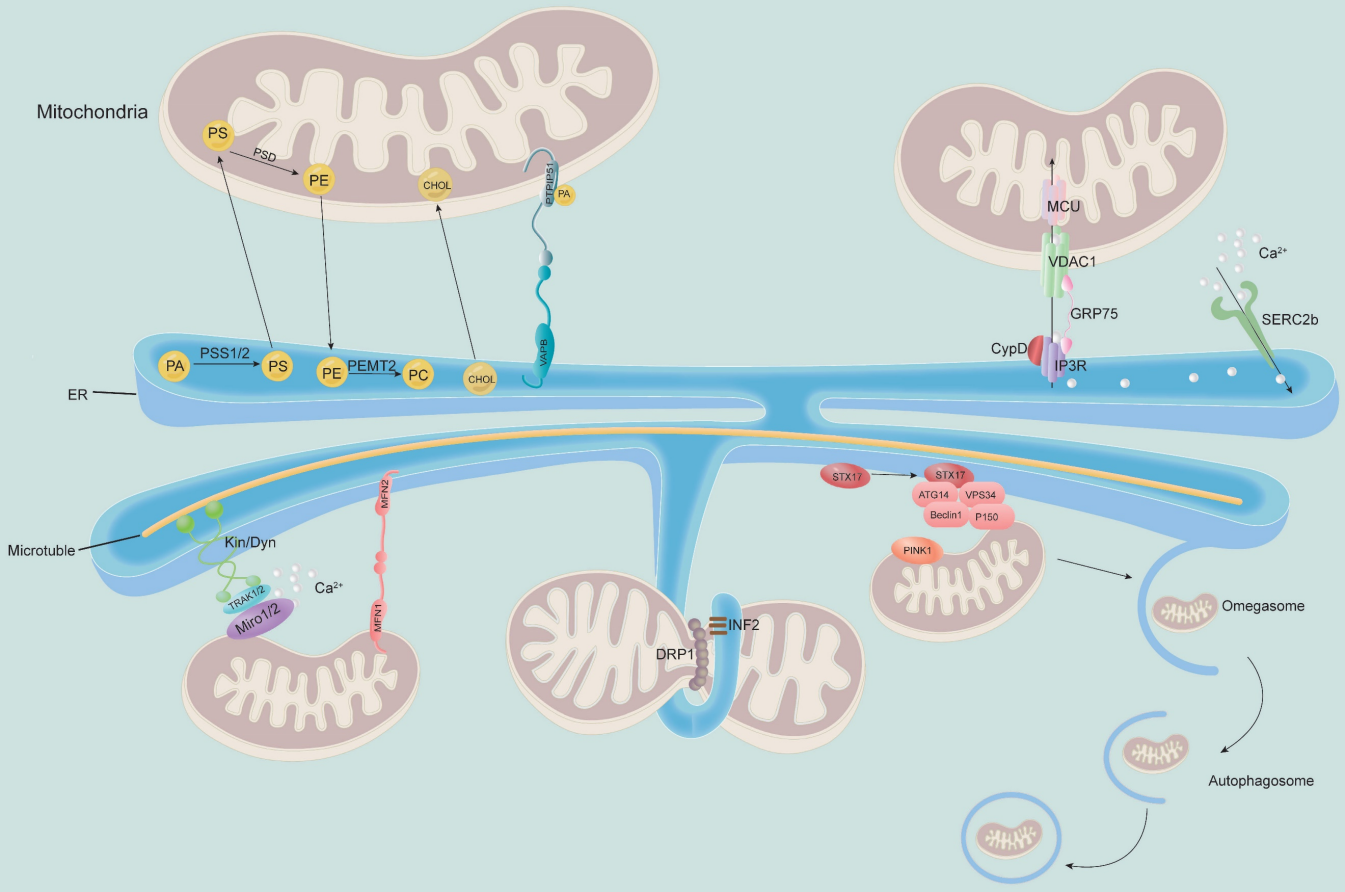
** MAM regulates the physiological function of mitochondria.** MAM regulates cellular lipid metabolism (top left) and calcium homeostasis (top right). The MAM is a key physical connection region for lipid metabolism, including phosphatidylcholine (PC) synthesis and cholesterol transport. In addition, MAM also participates in regulating calcium homeostasis through the IP3R-GRP75-VDAC1-MCU axis, which is an important channel for calcium transport from the ER to mitochondria (bottom). Meanwhile, MAM is considered a regulator of mitochondrial physiology: mitochondrial contraction occurs near the site of contact with the ER, which determines where mitochondrial fission occurs. Other proteins localized to MAM, such as STX17 and INF2, are involved in this process. Moreover, the ER remains attached to the mitochondria and travels along microtubules in the cell. MAM-resident STX17 binds ATG14 and recruits the class III PI3K complex to MAM, which contributes to the formation of autophagosomes.

MAM plays a key role in multiple physiological activities of cells, participating in lipid synthesis and transport, maintaining Ca^2+^ homeostasis, regulating mitochondrial fission and fusion, apoptosis and autophagy and other cellular processes (**Figure 2**).

### Lipid synthesis and transport

Accumulating evidence indicates that one of the major functions of MAMs is the regulation of biosynthesis and intermembrane transport of phospholipids 55. MAM forms a hydrophilic environment between the ER and mitochondria, which is conducive to the bidirectional noncystic transfer of lipids 38. Phosphatidylethanolamine (PE) and cardiolipin (CL) are essential phospholipids for mitochondrial respiratory function and can be synthesized in the mitochondria; however, their precursors and most of the mitochondrial phospholipids need to be synthesized in the ER before being transported to the mitochondria 56. The precursor of phosphatidylserine (PS), which is required for PE synthesis, is synthesized by phosphatidylserine synthase (PSS) in MAMs 34. In the study of Yang et. al, it was found that when PSS1 and PSS2 were knocked down, the content of PS was decreases, subsequently led to a change in the steady state of phosphatidylserine in the ER and diversion of lipid synthesis to triacylglycerol and diacylglycerol synthesis, thereby resulting in lipid accumulation 57. María Isabel et. al confirmed that MFN2 could selectively bind to PS and transfer it to mitochondria. Knockout of MFN2 reduced the expression of PSS1 and decreased the transfer of PS from the ER to the mitochondria, resulting in impaired phospholipid synthesis and ER stress 40. Moreover, overexpression of PSS1 promotes PS synthesis but cannot rescue the synthesis of PE 40. Additionally, Oxysterol-binding protein-related protein 5 (ORP5) and ORP8 also localize to the ER-mitochondria contact and interact with the intermembrane space bridging (MIB)/mitochondrial contact site and junctional junction organizing system (MICOS) complex and the OMM protein tyrosine phosphatase-interacting protein 51 (PTPIP51), mediating the transfer of PS from ER to mitochondria, and their depletion leads to defects in mitochondrial morphology and respiratory function 41, 42. Therefore, it is assumed that these functions of PS may be of great significance to explore the ectopic lipid deposition of kidney in DKD. However, whether the change of PSS itself at MAM directly triggers MAM dysfunction remains unclear, and it is also worth exploring in the future. CL is synthesized through a series of modifications of phosphatidic acid (PA), and recent studies have shown that the VAPB-PTPIP51 complex regulates the transfer of PA in MAMs 43. Moreover, many proteins related to lipid metabolism are enriched in MAMs, including diacylglycerol O-acyltransferase 2 (DGAT2), phosphatidylethanolamine N-methyltransferase 2 (PEMT2), fatty acid CoA ligase 4 (FACL4), cholesterol acyltransferase/sterol O-acyltransferase 1 (ACAT1/SOAT1), and PSS1/2 34, 58. Cholesterol likely reaches mitochondria through specialized MAM 59. The MAM-associated protein caveolin-1 (CAV1) has recently been shown to regulate ER-mitochondrial cholesterol transfer 45, and it regulates cholesterol efflux by binding to VDAC2. Inhibition of CAV1 is associated with abnormal intracellular accumulation of free cholesterol and reduced MAM physical extension and integrity 60.

### Ca^2+^ homeostasis

Intracellular Ca^2+^ homeostasis is mainly characterized by the balance and exchange of Ca^2+^ between the ER and mitochondria to maintain normal cell function 61. MAMs are hubs for Ca^2+^ signaling. The ER and mitochondria are important Ca^2+^ storage organelles, and the transfer of Ca^2+^ from the ER to the mitochondria relies on MAM-mediated physical contact sites between these two organelles 62. The ER is the largest intracellular Ca^2+^ pool 63, which performs its physiological function mainly through SERCA uptake and Ca^2+^ release by IP3R 46. The OMM protein voltage-dependent anion channel 1 (VDAC1) and the IMM protein mitochondrial calcium uniporter (MCU) are important proteins that take Ca^2+^ from the MAM gap into the mitochondrial matrix 64. The physical junction between the ER and mitochondria has a high concentration of Ca^2+^ microregions conducive to Ca^2+^ signal transduction 65. Various proteins in MAMs are related to Ca^2+^ signal transduction. IP3R⁃GRP75⁃VDAC1 is the major protein complex responsible for Ca^2+^ signaling between the ER and mitochondria 66. After activation, IP3R on the ER membrane releases Ca^2+^ from the ER lumen. With the assistance of GRP75, it is taken up by VDAC1 on the OMM, enters the mitochondrial matrix through the MCU of the IMM, and forms a Ca^2+^ signal flow in MAMs to play a biological role. In addition to Grp75, recent studies have shed light on other molecular partners at MAMs, such as cyclophilin D (CypD), which regulates ER-mitochondria crosstalk. CypD is a mitochondrial chaperone foldase and is sensitive to Ca^2+^ that interacts with the VDAC1-GRP75-IP3R complex at the interface of MAM and transfers Ca^2+^ from the ER to mitochondria in the heart and liver through IP3R1 and IP3R2 48, 67. Recent studies have shown that activation of CypD by high Ca^2+^ concentrations is one of the causes of oxidative stress and kidney injury in DKD 68, and a lack of CypD appears to exacerbate kidney injury in STZ-induced diabetic mice 69. However, whether MAM has an effect on this process needs to be addressed in future studies.

### Mitochondrial dynamics

Mitochondria are double-membrane organelles that regulate cellular metabolism and overall function. Mitochondrial dysfunction is recognized as an important contributor to DKD 70. Mitochondrial dynamics include mitochondrial fission, fusion and motility and mitophagy 71.

Mitochondrial fission is controlled by a group of motility-associated GTPase proteins, including DRP1 and its outer membrane receptors fission-1 (Fis1), mitochondrial fission factor (MFF), and mitochondrial dynamics proteins 49 and 59 (MiD49 and MiD59) 49. DRP1 possesses membrane contractile and severing capabilities and is the main driving force for performing mitochondrial and peroxisome fission 72; it increases ER-mitochondria interactions by promoting tubule formation in the ER 73. Binding of DRP1 to F-actin activates DRP1 GTPase activity, leading to the recruitment of DRP1 to preconstricted mitochondria to stimulate their fission 74. In the process of mitochondrial fission, DRP1 is recruited to the OMM, forming oligomeric complexes that surround, constrict and divide mitochondria 75. As DRP1 is normally cytosolic and lacks a domain that directly binds to membrane phospholipids, a collection of MFFs, MiD49 and MiD51, are recruited to the OMM through the receptor protein Fis1 76. These proteins are known to localize at the ER‐mitochondria interface. Several other MAM-associated molecules are also involved in mitochondrial fission. The ER-anchored isoform of the formin protein inverted formin 2 (INF2) mediates actin polymerization and facilitates mitochondrial DRP1 recruitment at ER-mitochondria intersections 77. It is a key step in mitochondrial fission, resulting in increased ER-mitochondria contacts, mitochondrial Ca^2+^ uptake, and mitochondrial fission 78. Mitochondrial Ca^2+^ uptake decreases after knocking out MCU, which leads to a decrease in mitochondrial fission 79. Overexpression of phosphofurin acidic cluster sorting protein 2 (PACS2) also suppresses mitochondrial fission and was reported to alleviate excessive mitochondrial fission by blocking mitochondrial recruitment of DRP1 in HK-2 cells cultured with high glucose 80. In addition, FUN14 domain-containing protein 1 (FUNDC1) aggregates in large quantities at the MAM during anoxia and promotes mitochondrial fission by binding to calcinetin (CANX) in response to anoxia 81.

The mitochondrial fusion process is divided into OMM fusion and IMM fusion 82. The OMM proteins MFN1 and MFN2, as well as the OMM protein optic atrophy 1 (OPA1), play major roles in mitochondrial fusion 51. Whereas MFN2 on the surface of the ER regulates mitochondrial connections to maintain the whole mitochondrial network, MFN1 on the surface of mitochondria plays a critical role in mitochondrial docking and fusion. The two proteins generate dimers and drive fusion of the OMM 83.

Mitochondrial motility is defined as transport along microtubules by opposing kinesin and dynein motors to regulate the distribution of mitochondria in the cytoplasm, which is essential to maintain the normal function of cells. Kinesin and dynein do not directly associate with the OMM but bind indirectly to the OMM proteins Miro1 and Miro2 via TRAK1 and TRAK2 59^.^ Current studies have shown that the role of Miro1/2 in mitochondrial movement is closely related to MAMs. It was reported that the yeast ortholog of Miro1 60, whereas loss of Miro1/2 alters mitochondria-ER communication 61. However, this binding requires a higher Ca^2+^ concentration. Due to the relatively low calcium affinity of Miro1/2, MAM is ideal for mitochondrial motility 60, 61^.^

Mitophagy is a selective autophagy process used to eliminate damaged mitochondria 84. The key regulators of mitophagy, PTEN-induced putative kinase 1 (PINK1) and Beclin1, both relocalize to MAMs during autophagy, which promotes the enhancement of ER-mitochondrial contact sites and the formation of autophagosome probes 52. Another mitophagy-associated protein, FUNDC1, was also shown to accumulate at the ER-mitochondrion interface during mitophagy by binding to ER-resident IP3R2 53. It was reported that MAM-resident syntaxin 17 can bind to the autophagosome to label autophagy related 14 and accumulate in the MAM until autophagosome formation is complete 85.

## The role of MAM in DKD

**Figure 3:**
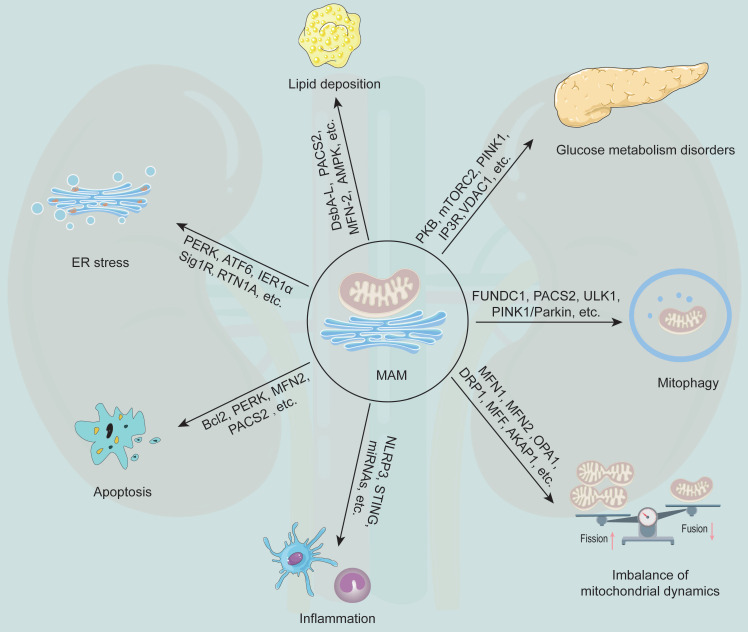
** The multifunction of MAM in DKD.** MAM is a special membrane contact site between the ER and mitochondria, and MAM-resident proteins play key roles in regulating various cellular processes that associated with the development of DKD, including glucose metabolism disorders, lipid deposition, ER stress, apoptosis, inflammation, mitochondrial dynamics imbalance, and mitophagy.

MAM plays a generalist role in the occurrence and development of DKD (**Figure 3**). Current studies have revealed that MFN2, DRP1, PACS2, PINK1, disulfide-bond A oxidoreductase-like protein (DsbA-L), nucleotide binding and oligomerization domain-like receptor family pyrin domain-containing 3 (NLRP3) and other MAM-related proteins are involved in regulating the initiation and development of various cellular processes, such as lipid metabolism, cell apoptosis, mitochondrial fission and fusion, and mitophagy (**Table 2**).

### Regulation of glucose metabolism

The main cause of T1D is the destruction of pancreatic β-cells, leading to deficient insulin production and secretion 97. Disruption of Ca^2+^ signaling at the MAM interface may be a key feature of glucotoxicity-mediated β-cell dysfunction 98. T2D is characterized by insulin resistance and compensatory hyperinsulinemia, which results in the development of microvascular complications, including DKD 99. Podocytes are insulin-sensitive renal cells, and thus, insulin resistance is more likely to cause kidney damage 100. Insulin-stimulated podocytes rapidly transport glucose to the cytoplasm to provide energy for maintaining their actin skeleton and normal filtration function. A growing body of evidence has supported that MAM integrity is required for insulin signaling 101. It may mediate insulin resistance by regulating Ca^2+^ transduction, lipid metabolism, mitochondrial function, ER stress, etc. 102. The proteins mTORC1 and protein phosphatase 2A (PP2A) located in MAMs play core roles in regulating podocyte insulin resistance 86. Another MAM protein associated with insulin resistance, PINK, has been found to be deficient, significantly increasing albumin permeability and hindering glucose uptake in podocytes, which supports the crucial role of PINK1 in maintaining insulin signal transduction and podocyte permeability 54. Therefore, MAM might participate in regulating insulin resistance in DKD; however, further studies are needed to confirm its potential.

### Regulation of lipid metabolism

Lipid metabolism disorder is a key pathogenic feature of DKD 103. High glucose induces lipid deposition in the kidney, and lipid deposition in nonadipose tissues such as the liver and kidney are called ectopic fat deposition (EFD) 104. Lipid deposition has been found to be mainly distributed in tubules and glomeruli in animal models of DKD 105, 106. EFD in renal tissue is one of the main factors leading to renal fibrosis and CKD 107. It was reported that MAMs were significantly reduced in renal tissues of DKD patients, and the expression of MAM-related proteins such as DsbA-L, PACS2 and MFN2 was reduced and inversely correlated with blood lipid levels and the degree of renal lipid deposition, suggesting that a disruption of MAM integrity may lead to renal lipid deposition in DKD 108. AMP-activated protein kinase (AMPK) is an important protein involved in the formation of MAMs 109. The study by Chen et al. found that inhibition of DsbA-L expression aggravated lipid deposition in DKD, and activation of the AMPK pathway is a potential mechanism by which DsbA-L plays a role in renal lipid deposition 110. Zhao et al. found that diabetic mice with proximal tubule PACS2-specific knockout had more severe tubular damage and proteinuria than controls, which was accompanied by increased lipid synthesis, decreased cholesterol uptake and efflux, and lipid deposition in renal tubules 111, indicating that PACS2 is a major regulator in lipid-related kidney lesions in DKD. These studies suggested that MAM-related proteins played key roles in regulating lipid deposition in DKD, but more research is needed to elucidate the molecular mechanism of MAM involvement in EFD in DKD.

### Regulation of inflammation

Persistent inflammation in the circulatory system and kidney tissue is an important pathophysiological basis in the development of DKD 112. The role of MAMMAMs in inflammation is related to NLRP3 inflammasome assembly and activation 113. Inactivated NLRP3 is located in the ER membrane and cytoplasm, while activated NLRP3 can combine with apoptosis-associated speck-like protein containing a CARD (ASC) in MAM to form inflammasomes 114, 115. Recently, Yang et al*.* found that DsbA-L attenuated NLRP3-mediated renal inflammatory injury by promoting AMPK phosphorylation in DKD 88, which suggested that MAM acted as an inflammatory regulator through NLRP3 in DKD. In addition, as a key mediator of innate immunity, stimulator of interferon genes (STING), which can recognize exogenous and endogenous DNA in cells, has been shown to be present in the ER and MAM 116. A new discovery by Feng et al*.* showed that the enhanced inflammatory response caused by HIV virus infection in podocytes might be a key accelerator for the progression of DKD 117. Coincidentally, STING is known to bind mitochondrial antiviral signaling protein (MAVS) on MAM, thereby increasing the interferon response to viral infection 118. Depending on the unique localization of STING on the MAM, it has been suggested that STING-MAM crosstalk has a nonnegligible effect on the immune response 119. Additionally, as a key player in metabolic inflammation, STING induces podocyte injury in* db/db* mice 87. Therefore, STING may regulate the inflammation progression of DKD through MAMs. In addition, it has also been found that MAMs are subcellular sites of specific miRNAs 120. In this study, Wang et al. found that MAMs are rich in inflammatory response miRNAs in human and rat brains, including miR-146a, miR-142-3p and miR-142-5p 120. Coincidentally, miR-146, miR-142-3p, and miR-142-5p have been shown to play a regulatory role in the progression of DKD inflammation 121-123. Therefore, there is a possibility that MAMs participate in the inflammatory response of DKD through miRNAs. However, due to the highly conserved nature of MAMs, more studies are needed to confirm whether MAMs enrich the expression levels of inflammatory-responsive miRNAs in DKD.

### Regulation of ER stress

The ER is a major site for protein synthesis, folding, processing, and quality control 124. The imbalance between ER protein-folding load and capacity under various physiological and pathological conditions results in ER stress 125. However, sustained stress stimulation promotes the switch of the adaptive unfolded protein response (UPR) into proapoptotic signals 123. ER stress is mediated by ER-localized sensor protein kinases RNA-dependent protein kinase (PKR)-like ER kinase (PERK), activating transcription factor 6 (ATF6) and inositol-requiring enzyme 1α (IRE1α), which are retained in their inactive states by interacting with GRP78 126. When ER stress occurs, they are separated from GRP78 to activate downstream signaling pathways and then restore ER homeostasis by reducing protein translation and promoting chaperone production 127. Various ER partners involved in protein folding, including Bid, calnexin, calreticulin and sigma-1 receptor (Sig1R), are located in the MAM 128. IRE1α was reported to be expressed in the MAM and to associate with Sig1R during ER stress 129. PERK is a sensor for the UPR and has been identified as a key MAM component 130. MFN2 is an upstream modulator of PERK 131. It was found that reduced MFN2-PERK interaction was accompanied by decreased MFN2 expression and activation of all three UPR pathways in DKD 89. The ER-resident protein reticulon-1A (RTN1A) is known to mediate podocyte and tubular cell injury in DKD by modulating ER stress 132, 133. A recent study found that overexpression of RTN1A exacerbated ER stress in DKD by modulating MAM 16. This is mainly because RTN1A interferes with the interaction of mitochondrial hexokinase-1 and VDAC1, leading to the activation of apoptosis and inflammatory pathways 16. In summary, the involvement of MAM in DKD is substantial, as it contributes to ER stress, and PERK, MFN2, Sig1R, and RTN1A are known to be integral to this process.

### Regulation of cell apoptosis

Apoptosis is believed to be an important cause of DKD 134. Correspondingly, this process is closely related to MAM-mediated Ca^2+^ regulation. B-cell lymphoma 2 (Bcl2) family proteins are key members in mediating Ca^2+^ transport from the ER to mitochondria and regulating apoptosis 135. Bcl2 is predominantly located in the resting ER and translocates to the MAM and mitochondria upon induction of apoptosis 136. As an extensively studied anti-apoptotic protein, Bcl2 is often used as a marker to detect apoptosis in DKD. Its expression was significantly decreased in DKD patients 90. Recent studies have shown that TOM20 promotes the transfer of Bcl2 from the ER to the MAM and mitochondria during apoptosis induction 136. Many multifunctional MAM-associated proteins have been found to play an antiapoptotic role in DKD. MFN2 exerts mitochondrial protective and antiapoptotic effects by maintaining the number and integrity of MAMs and inhibiting the activation of the PERK pathway 89. Meanwhile, several MAM-associated proteins are also involved in apoptosis by interacting with MFN2. For example, DsbA-L plays an antiapoptotic role in DKD by promoting the expression of MFN2 and maintaining the integrity of MAMs 137. However, these beneficial effects were partially blocked by overexpression of FATE-1, a MAM uncoupling protein 137. PACS2 is also a key molecule required to maintain MAM homeostasis in diabetic tubular injury. Xue et al. observed that PACS2 deficiency reduced the integrity of MAMs and exacerbated renal cell apoptosis in diabetic mice 91. Taken together, these studies strongly support that MAM plays an important role in regulating cell apoptosis in DKD.

### Regulation of mitochondrial fission and fusion

As an energy-intensive organ, the kidney is the second-largest relative to the heart in mitochondrial abundance 138. Mitochondrial dysfunction has a serious impact on kidney function. Furthermore, changes in the regulation of mitochondrial dynamics and ultrastructure precede the development of albuminuria and renal histological changes in diabetes, and these mitochondrial changes evolve as DKD progresses 139. Therefore, it is of great significance to study the markers related to mitochondrial dysfunction in DKD. Mitochondria are highly dynamic organelles that continually maintain cell survival and bioenergetics through fission, fusion, mitophagy and other mitochondrial quality control processes 140. The balance of mitochondrial fusion and fission is necessary to maintain the normal shape and function of mitochondria under physiological conditions 141. It has been reported that MAM-related proteins, such as MFN1, MFN2, OPA1 and DRP1, are involved in regulating mitochondrial fission and fusion. For example, under high glucose condition, the expression of the mitochondrial fusion markers MFN1, MFN2 and OPA1 was decreased, while the activity of the fission marker DRP1 was increased in human podocytes 142. Xiao et al. found that the mitochondrial fission of podocytes increased under high glucose conditions, while the expression of MFF was upregulated. After MFF expression inhibition, the podocyte survival rate was significantly reduced 92. It is suggested that the increase in mitochondrial number caused by MFF-mediated mitochondrial fission may be a response mechanism to mitochondrial overload and protect podocytes in the short term. Li et al. reported that the translocation of A-kinase-anchored protein 1 (AKAP1) to MAMs was increased, which promoted podocyte mitochondrial fission by regulating DRP1 phosphorylation and its subsequent mitochondrial translocation 93.

### Regulation of autophagy

Autophagy is considered an evolutionarily conserved cellular process responsible for digesting or recycling organelles and long-lived proteins to maintain cellular homeostasis 143. Accumulation of fragmented mitochondria has been found in the kidney in both humans and animals with DKD 144, 145, suggesting that the mitochondrial clearance machinery may be impaired as the kinetics shift. The OMM protein FUNDC1, a novel MAM protein, is enriched at MAM through interaction with the ER-resident protein CANX under hypoxic conditions. During mitochondrial phagocytosis, it dissociates from CANX and preferably recruits DNM1L/DRP1 to drive mitochondrial fission in response to hypoxic stress 81. FUNDC1 has been shown to be involved in podocyte mitophagy in DKD 94. Furthermore, Li et al. revealed that the overexpression of PACS2 in HK-2 cells blocked mitochondrial recruitment of DRP1 and alleviated excessive mitochondrial fission induced by high glucose condition, subsequently restoring MAM integrity and enhancing mitophagy 80. PINK1/Parkin and ULK1 are considered mitophagy-related genes in DKD 95, 96 and have been reported to localize to MAMs during mitophagy 52, 146.

### Potential role of MAM in DKD

In view of the important role of MAM in kidney injury, it is possible to develop MAM-related proteins as therapeutic targets for DKD. New research has found that AMPK, MFN2, PACS2 and DsbA-L play essential roles in regulating glucose and lipid metabolism and thus can be used as promising targets for the treatment of DKD. For example, curcumin inhibited renal lipid accumulation and oxidative stress through the AMPK and Nrf2 signaling pathways in DKD mice 147.

As an important protein mediating ER stress in DKD, targeting PERK for DKD has been extensively studied. Yuan et al. found that resveratrol treatment reduced the phosphorylation of PERK and regulated ER stress in the kidneys of diabetic rats 148. Additionally, Tian et al. found that emodin attenuates ER stress-induced podocyte apoptosis by inhibiting the PERK signaling pathway in DKD 149. In addition, NLRP3 has attracted wide attention as a target for inflammation regulation. Curcumin, the major bioactive compound of turmeric, exerts antifibrotic effects in DKD by inhibiting the NLRP3 inflammasome 146. Dapagliflozin, an SGLT2 inhibitor, has been shown to reduce renal injury even in the absence of diabetes through inhibition of the NLRP3 inflammasome, protecting against kidney fibrosis 150. In addition, Bcl2, MFN2, PACS2 and Beclin1 are potential targets for modulating apoptosis. It was reported that wogonin protects glomerular podocytes by targeting Bcl2-mediated autophagy and apoptosis in DKD 90. Curcumin has also been shown to regulate apoptosis in DKD. It inhibited podocyte apoptosis and accelerated autophagy in diabetic nephropathy by regulating Beclin1/UVRAG/Bcl2^151^. FUNDC1 and Parkin are potential targets for regulating mitophagy in DKD. Metformin is a classic antidiabetic drug that inhibits the expression of Parkin and mitophagy by activating PP2A and inhibiting NF-κB, which protects renal epithelial cells from high glucose stimulation *in vitro* 152.

Due to the imbalance of mitochondrial division and fusion in DKD, inhibition of DRP1 function may be a new therapy for diabetic albuminuria 153. A novel mitochondrial target peptide, SS31, which targets mitochondrial fission and fusion, has been found to reduce mitochondrial fragmentation by inhibiting the expression of DRP1 and increasing the expression of MFN1, thus preventing STZ-induced kidney injury in mice 154. In addition, a recent study developed a covalent compound, mitochondrial division inhibitor (MIDI), which functionally mimics DRP1 knockdown to inhibit mitochondrial fission but prevents the recruitment of DRP1 to mitochondria, thereby preventing mitochondrial fission 155.

## Outlook

The interactions between organelles are essential for eukaryotes to maintain normal physiological functions. It is widely acknowledged that the mitochondria and ER are significant players in the development of DKD 13, 14. The MAM serves as a platform for facilitating communication between the ER and mitochondria, enabling the rapid exchange of biomolecules to maintain cellular health and function 57. MAM dysfunction leads to the development of various diseases through multiple pathways. Studies have demonstrated that MAM plays a crucial role in maintaining Ca^2+^ homeostasis, lipid synthesis and transport, and metabolism, as well as regulating autophagy 156 and monitoring the morphology and function of the ER and mitochondria. In addition, as a dynamic structure, MAM also provides a platform for numerous enzymes to operate, and miRNAs are also highly concentrated in MAM 125.

Recent studies have revealed that MAM plays a crucial role in several processes related to DKD, such as Ca^2+^ overload, lipid metabolism, apoptosis, mitochondrial fission and fusion, and mitophagy 80, 104, 137. Based on the present studies, we speculated that MAM contributed to the progression of DKD via insulin resistance and inflammation 52, 54, 88, 113. Several MAM-related proteins have been deeply investigated in DKD, such as DsbA-L, PACS2, DRP1, and MFN2 91, 93. All of these proteins play crucial roles in different aspects of cell function and are considered promising targets for DKD treatment. Numerous drugs have been discovered to target MAM, which are beneficial in DKD treatment. For example, curcumin targets AMPK, NLRP3, and Bcl2 to lower ER stress, inflammation and apoptosis in DKD 146, 147, 151. Metformin, dapagliflozin, wogonin, resveratrol, and SS31 are also effective in treating DKD by targeting MAM 90, 150, 152, 154. However, targeting MAM-related proteins does not necessarily alter the MAM itself. For example, a study by Maya et al. showed that although metformin treatment improves blood glucose levels and insulin sensitivity to some extent in diabetic mice, it does not prevent the changes in MAM Ca^2+^ coupling in cardiac muscle cells and the resulting heart dysfunction induced by T2D 157. On the other hand, Wei et al. found that activation of transient receptor potential cation channel subfamily V member 1 (TRPV1) by capsaicin alleviated mitochondrial dysfunction caused by high glucose in podocytes and was accompanied by a reduction in MAM formation and decreased Ca^2+^ transport from the ER to mitochondria 158. This is because the transient influx of Ca^2+^ mediated by TRPV1 reduced the transcription of the key molecule FUNDC1, which was crucial for MAM formation 158. These findings strongly support MAM as a potential target for the treatment of DKD.

Although some MAM-related metabolic regulators and signaling pathways are involved in regulating DKD, there remain several unclear questions. What are the essential elements for preserving MAM structure and function? What is the precise regulatory mechanism by which MAM contributes to DKD? Notably, there is still no specific molecules to dynamically trace ER-mitochondria interaction, which makes it difficult to conduct in-depth study on the key role of MAM in DKD. In addition, the kidney is composed of various cell types, including podocytes, mesangial cells, endothelial cells, and renal tubular cells, which may have distinct functions associated with MAM. Therefore, investigating the MAM of different cell lines is crucial to understanding its role in various diseases. It is noteworthy that MAM is a transient structure, and its state may vary during different stages of disease. Therefore, further extensive research is necessary to fully explain the multifunctional attributes of MAM dysfunction in DKD.

### Data availability

The data used to support the findings of this study are included within the paper.

### Author Contributions

Yong Liu, Qi Feng and Zhangsuo Liu designed the manuscript. Yong Liu, Qi Feng, Dongwei Liu and Zhangsuo Liu contributed to writing the manuscript. Yong Liu, Yingjin Qiao, Dongwei Liu, Shaokang Pan, Jingfang Chen, Zihui Mao, Kaidi Ren, Yang Yang, Qi Feng and Zhangsuo Liu reviewed and revised the manuscript. All authors have seen and approved the final version of the manuscript being submitted.

## Figures and Tables

**Table 1:** Structure and function-related proteins in MAM

Function types	Proteins	Abbreviation	Relevant functions in MAM
Lipid metabolism	Fatty acid CoA ligase 4	FACL4	Immobilize fatty acids on CoA 37
	Acy1-Coenzyme A-cholesterol acyltransferase	ACAT	Synthesize cholesteryl esters 38
	Phosphatidylethanolamine N-methyltransferase 2	PEMT2	Convert PE to PC in ER 39
	Mitofusin-2	MFN2	Transfer PS to mitochondria from ER 40
	Oxysterol-binding protein-related protein 5	ORP5/8	Mediating the transfer of PS from ER to mitochondria 41, 42
	Protein tyrosine phosphatase-interacting protein 51	PTPIP51	Regulates the transfer of PA in MAM 43
	Diacylglycerol O-acyltransferase 2	DGAT2	Catalyzes triglyceride synthesis 44
	Phosphatidylserine synthase 1 and 2	PSS1/2	Synthesize PS 34
	Caveolin-1	CAV1	Regulate cholesterol efflux 45
Ca^2+^ hemostasis	Inositol1,4,5-trisphosphate receptor	IP3Rs	Major calcium channels in ER 46
	Voltage-dependent anion channel 1	VDAC1	Calcium uptake channels in mitochondria 47
	Glucose regulated protein 75	GRP75	Connects IP3R and VDAC to form VDAC1/GRP75/IP3R1 channel complex 47
	Cyclophilin D	CYPD	A partner of the IP3R1-GRP75-VDAC1 complex and changes the MAM spatial structure 48
	Sarco/endoplasmic reticulum Ca^2+^ ATPase	SERCA2b	Acts as an important pump involved in Ca^2+^ transport into ER 46
Mitochondrial dynamics	Dynamin-related protein 1	DRP1	Control mitochondrial fusion 49
	Inverted formin 2	INF2	Driving initial mitochondrial constriction 50
	Mitofusin-2	MFN2	Mediate mitochondrial fusion 51
	Optic atrophy 1	OPA1	Mediate mitochondrial fusion 51
	PTEN-induced putative kinase 1	PINK1	Mediates mitophagy 52
	FUN14 domain-containing protein 1	FUNDC1	Mediates mitophagy 53
Insulin signaling	Protein kinase B	PKB	Maintains insulin signal transduction 36
	mammalian target of rapamycin complex (mTORC)	mTORC	Maintains insulin signal transduction 36
	PTEN-induced putative kinase 1	PINK1	Maintains insulin signal transduction 54

**Table 2:** MAM-associated key components participate in DKD

Functions in DKD	Proteins in MAMs
Regulate glucose metabolism	PINK1^54^, mTORC1^86^, PP2A^86^
Regulate lipid metabolism	DsbA-L, PACS2, MFN2^93^, AMPK 95
Regulate inflammation	STING^87^, NLRP3^88^
Regulate ER stress	PERK, MFN2^89^, RTN1A, VDAC1^16^
Regulate renal cell apoptosis	Bcl2^90^, PERK, MFN2^79^, PACS2^91^
Regulate mitochondrial fission and fusion	MFN1, MFN2, OPA1, DRP1^136^, MFF^92^, AKAP1^93^
Regulate mitophagy	FUNDC1^94^, PINK1/Parkin^95^, ULK1^96^

## Abbreviations

MAM: Mitochondria-associated endoplasmic reticulum membrane
ER: Endoplasmic reticulum
MSC: Membrane contact site
DKD: Diabetic kidney disease
DM: Diabetes mellitus
T1D: Type 1 diabetes
T2D: Type 2 diabetes
ESRD: End-stage renal disease
GFR: Glomerular filtration rate
DGAT2: Diacylglycerol O-acyltransferase 2
PEMT2: Phosphatidylethanolamine N-methyltransferase 2
FACL4: Fatty acid CoA ligase 4
ACAT1/SOAT1: Cholesterol acyltransferase/sterol O-acyltransferase 1
PSS1/2: Phosphatidylserine synthase 1/2
PS: Phosphatidylserine
PE: Phosphatidylethanolamine
PA: Phosphatidic acid
CL: Cardiolipin
OMM: Outer mitochondrial membrane
IMM: Inner mitochondrial membrane
PC: Phosphatidylcholine
PEMT2: Phosphatidylethanolamine N-methyltransferase 2
PTPIP51: Protein tyrosine phosphatase-interacting protein 51
CAV1: Caveolin-1
IP3R: Inositol 1,4,5-trisphosphate receptor
GRP75: Glucose regulated protein 75
VDAC1: Voltage-dependent anion channel 1
MCU: Mitochondrial calcium uniporter
CypD: Cyclophilin D
CANX: Calcinetin
MFN2: Mitofusin 2
PKB: Protein kinase B
mTORC: Mammalian target of rapamycin complex
DRP1: Dynamin-related protein 1
Fis1: Fission-1
MFF: Mitochondrial fission factor
MID: mitochondrial dynamics proteins 49
OPA1: Optic atrophy 1
INF2: Inverted formin 2
PACS2: Phosphofurin acidic cluster sorting protein 2
PP2A: Protein phosphatase 2A
PINK1: PTEN-induced putative kinase 1
DsbA-L: Disulfide-bond A oxidoreductase-like protein
FUNDC1: FUN14 domain-containing protein 1
EFD: Ectopic fat deposition
NLRP3: Nucleotide binding and oligomerization domain-like receptor family pyrin domain-containing 3
ASC: Apoptosis-associated speck-like protein containing a CARD
MAVS: Mitochondrial antiviral signaling protein
STING: Stimulator of interferon genes
AMPK: AMP-activated protein kinase
UPR: Unfolded protein response
Sig1R: Sigma-1 receptor
PERK: RNA-dependent protein kinase (PKR)-like ER kinase
ATF6: Activating transcription factor 6
IRE1α: Inositol-requiring enzyme 1α
RTN1A: Reticulon 1A
AKAP1: A-kinase anchoring protein 1
Bcl2: B-cell lymphoma 2
MIDI: Mitochondrial division inhibitor
WASF3: Wiskott-Aldrich syndrome protein family member 3
ATAD3: ATPase family AAA Domain-containing protein 3
MITOL: Mitochondrial ubiquitin ligase
MOSPD2: Motile sperm domain-containing protein 2
